# Skin deep

**DOI:** 10.7554/eLife.21506

**Published:** 2016-10-14

**Authors:** Aitor Casas-Sánchez, Álvaro Acosta-Serrano

**Affiliations:** 1 Department of Parasitology, Liverpool School of Tropical Medicine, Liverpool, United Kingdom; 1 Department of Parasitology, Liverpool School of Tropical Medicine, Liverpool, United KingdomAlvaro.Acosta-Serrano@lstmed.ac.uk; 2 Department of Vector Biology, Liverpool School of Tropical Medicine, Liverpool, United Kingdom

**Keywords:** *Trypanosoma brucei gambiense*, Human African Trypanosomiasis, skin, reservoir, transmission, tsetse fly, Human, Mouse, Other

## Abstract

Trypanosome parasites are hiding in human skin, a discovery that may undermine efforts to eliminate sleeping sickness by 2020.

**Related research article** Capewell P, Cren-Travaillé C, Marchesi F, Johnston P, Clucas C, Benson RA, Gorman TA, Calvo-Alvarez E, Crouzols A, Jouvion G, Jammoneau V, Weir W, Stevenson ML, O'Neill K, Cooper A, Swar NK, Bucheton B, Ngoyi DM, Garside P, Rotureau B, MacLeod A. 2016. The skin is a significant but overlooked anatomical reservoir for vector-borne African trypanosomes. *eLife* **5**:e17716. doi: 10.7554/eLife.17716

Human African trypanosomiasis – also known as sleeping sickness – is a potentially fatal disease, which currently affects ~3,500 people in sub-Saharan countries, mostly in the Democratic Republic of the Congo (Franco et al., 2014). The disease is caused by parasites called African trypanosomes and is spread by tsetse flies. Controlling these biting insects, combined with surveillance and treatment, reduces the impact of outbreaks of the disease (Lehane et al., 2016), and the World Health Organisation (WHO) hopes to eliminate sleeping sickness by 2020. This target has, in part, been encouraged by the success of surveillance efforts that rely partly on detecting trypanosomes in human blood. Now, in eLife, Annette MacLeod, Brice Rotureau and colleagues – including Paul Capewell of the University of Glasgow and Christelle Cren-Travaillé of the Institut Pasteur as joint first authors – report results that suggest that this target might be overambitious (Capewell et al., 2016).

*Trypanosoma brucei gambiense* – the parasite responsible for most cases of sleeping sickness – has always been regarded as a blood-dwelling parasite. The blood might be an inhospitable environment for most parasites, but *T. brucei* survives because it outwits the human immune system by constantly changing the make-up of its outer coating via a process called antigenic variation (Mugnier et al., 2016).

Trypanosomes exist as two different forms in human blood: “slenders” that replicate and undergo antigenic variation; and smaller “stumpies” that do not undergo antigenic variation but are pre-adapted to survive in the tsetse fly (MacGregor et al., 2011, Dyer et al., 2013). In chronic infections, the parasites can also cross the blood-brain barrier and cause a number of potentially fatal neurological disorders, including the disrupted sleep patterns that give the disease its common name.

Capewell et al. – who are based in Glasgow, Paris, Montpellier and Kinshasa – now report that the skin is a reservoir for *T. brucei*. In fact, they found many trypanosomes dwelling in the skin of infected animals and people, even when none were detected in the bloodstream.

It was known that trypanosomes migrate into other tissues and organs in the body, but no one had reported that they could invade and spread systemically throughout the surface of the body before. Nevertheless, this is exactly what Capewell et al. observed after they injected trypanosomes into the abdominal cavity of mice. The mice developed bloodstream infections within a few days and the trypanosomes were detected in patches of the skin after as little as 12 days and persisted throughout the infection. This suggests that the parasites spread from the blood into the skin. Similar results were observed when the mice were exposed to bites from infectious tsetse flies.

Next, Capewell et al. demonstrated that these skin-dwelling parasites were viable, and that slender forms in the skin could successfully develop into stumpy forms. In fact, when tsetse flies fed on mice with trypanosomes in their skin but not their blood, the flies became infected (Figure 1). This strongly suggests that skin-dwelling parasites can contribute to the transmission of the disease.

**Figure 1.**
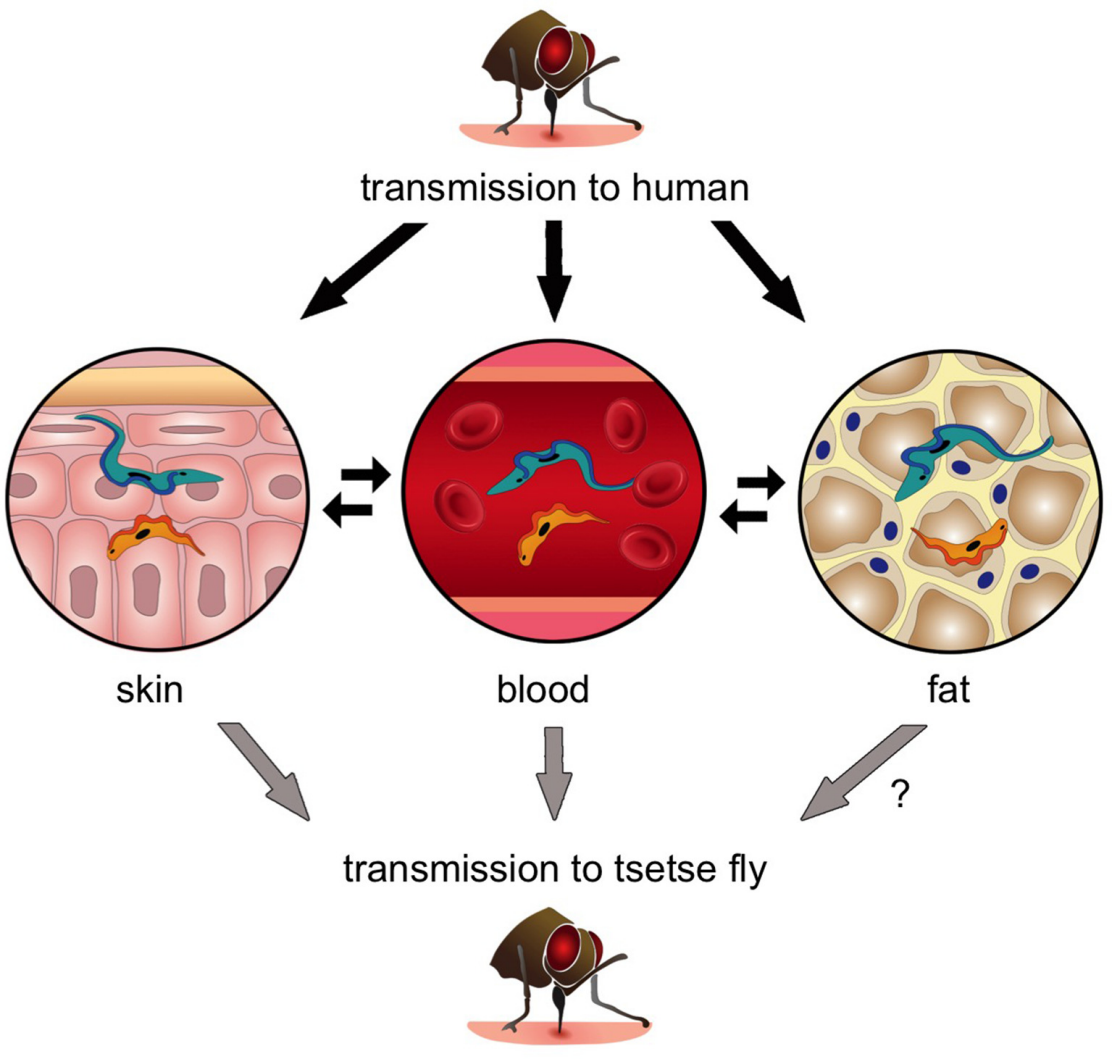
The transmission of *Trypanosoma brucei* from the tsetse fly to a human and back again. When an infected tsetse fly (top) bites a human it injects saliva that contains infectious trypanosomes into the skin. These parasites quickly transform into the “slender” form (blue), which can either go into the bloodstream or remain in the skin and nearby fat (i.e. the subcutaneous adipose). Once the trypanosomes become established in any of these tissues, they can transform into the “stumpy” form (orange) that can re-infect another tsetse fly (grey arrows). Transmission from fat to tsetse is plausible, but has not been demonstrated experimentally and is therefore labelled with a question mark. Parasite reservoirs might be less exposed to the immune system in skin or fat and may play an important role in transmitting sleeping sickness, and in maintaining the trypanosome infection in asymptomatic individuals.

To determine if people from an area where sleeping sickness is endemic harboured trypanosomes in their skin, Capewell et al. conducted an extensive analysis of over a thousand archived human skin biopsies. The archive had been collected in the Democratic Republic of the Congo as part of a screening programme for another disease, river blindness. Several (~0.5%) of these biopsies contained trypanosomes, and strikingly, these came from individuals without a history of sleeping sickness. Given that that these parasites only appear to infect patches of the skin, it is likely that the percentage of asymptomatic individuals carrying trypanosomes somewhere in their skin will be much higher.

The findings of Capewell et al. also complement two other papers published earlier this year. The first reported that *T. brucei* has a life stage that uniquely invades fat tissue (Trindade et al., 2016), while the second showed that trypanosomes spread by tsetse bites also persist in the skin around the bite and remain transmissible to tsetse flies (Caljon et al., 2016).

The results of Capewell et al. raise two fundamental questions related to the possible elimination of sleeping sickness: first, do available anti-trypanosomal drugs kill skin-dwelling parasites? Second, is sleeping sickness really spread from human to human, or is it spread from other animals that carry hidden trypanosomes in their skin and fat tissue? The answers to these questions remain unknown, but the detection of infectious skin-dwelling trypanosomes could explain why sleeping sickness has persisted in some areas, despite treatment and active surveillance. If confirmed, this discovery may lead to the potential introduction of a non-invasive test to detect trypanosomes in human skin. In the broader sense, it may also reset the WHO’s elimination clock because many more people might be carrying the transmissible parasites than previously predicted.
